# Myelin basic protein and TREM2 quantification in the CSF of patients with Multiple System Atrophy and other Parkinsonian conditions

**DOI:** 10.1007/s00415-024-12747-w

**Published:** 2024-12-12

**Authors:** Fabian Maass, Sezgi Canaslan, Christoph van Riesen, Peter Hermann, Matthias Schmitz, Claudia Schulte, Kathrin Brockmann, Matthis Synofzik, Mathias Bähr, Inga Zerr

**Affiliations:** 1 Department of Neurology, University Medical Center Göttingen, Robert-Koch-Str. 40, 37075 Göttingen, Germany; 2 German Center for Neurodegenerative Diseases (DZNE), Göttingen, Germany; 3 Hertie Institute for Clinical Brain Research and Center of Neurology, Department of Neurodegenerative Diseases, University of Tübingen, Tübingen, Germany; 4 German Center for Neurodegenerative Diseases (DZNE), Tübingen, Germany

**Keywords:** Biomarker, Myelin basic protein, Cerebrospinal fluid, Multiple system atrophy, TREM2

## Abstract

**Background:**

It is well known that myelin disruption and neuroinflammation are early and distinct pathological hallmarks in multiple system atrophy (MSA) as well as in idiopathic Parkinson’s disease and in other atypical Parkinsonian syndromes. The objective of this study was to assess the value of non-neuronal biomarker candidates that reflect myelin disruption and neuroinflammation.

**Methods:**

Myelin basic protein (MBP) and the soluble form of TREM2 were quantified in a comprehensive movement disorder cohort from two different neurological centers, comprising a total of 171 CSF samples. Commercially available ELISA systems were employed for quantification.

**Results:**

The results of the MBP analysis revealed a significant increase in cerebrospinal fluid (CSF) MBP levels in all atypical Parkinsonian conditions compared to PD. This differentiation was more pronounced in the MSA-c subtype compared to MSA-p. Receiver operating characteristic (ROC) analysis revealed a significant discrimination between PD and MSA (*p* = 0.032, AUC = 0.70), PD and DLB (*p* = 0.006, AUC = 0.79) and PD and tauopathies (*p* = 0.006, AUC = 0.74). The results of the TREM2 analysis demonstrated no significant differences between the PD and atypical Parkinsonian groups if not adjusted for confounders. After adjusting for age, sex, and disease duration, the PD group exhibited significantly higher TREM2 levels compared to the DLB group (*p* = 0.002).

**Conclusions:**

In conclusion, MBP, but not TREM2, is elevated in the CSF of not only MSA but in all atypical Parkinsonian conditions compared to idiopathic Parkinson’s disease. This highlights the value of the evaluation of myelin/oligodendrocyte-associated markers in neurodegenerative movement disorders.

**Supplementary Information:**

The online version contains supplementary material available at 10.1007/s00415-024-12747-w.

## Introduction

Multiple system atrophy (MSA) is a rare sporadic neurodegenerative disorder that is primarily characterized by autonomic dysfunction in conjunction with cerebellar symptoms or Parkinsonism. Depending on the location of the pathological process in the brain, two distinct motor subtypes are commonly distinguished: the cerebellar subtype (olivopontocerebellar atrophy; MSA-c) and the Parkinsonian subtype (striatonigral degeneration; MSA-p) [1].

Similar to Parkinson's disease (PD) and Dementia with Lewy bodies (DLB), MSA can also be classified as a synucleinopathy due to the accumulation of misfolded alpha-synuclein in the central nervous system (CNS). In contrast to the classical Lewy-body diseases PD and DLB, this accumulation is primarily observed in oligodendrocytes (glial cytoplasmic inclusions, or GCI) and only to a lesser extent in neurons [2]. Pathological changes besides neuronal degeneration can be found early in MSA, even before the occurrence of pathological alpha-synuclein aggregates. For instance, redistribution of the myelin protein TPPP/p25, myelin lipid dysregulation, as well as a pathological swelling of the oligodendroglial somas, can be found early in MSA preceding alpha-synuclein aggregation [3]. Despite the evident significance of these early, non-neuronal disease mechanisms, the majority of biomarker research for MSA has been concentrated on alpha-synuclein, in addition to different markers of neuronal degeneration and catecholamines [4]. The evaluation of oligodendrocyte and/or myelin-associated biomarker targets has been largely overlooked in MSA and atypical Parkinsonian syndromes, such as Dementia with Lewy bodies (DLB), or in tauopathies, including progressive supranuclear palsy (PSP) and corticobasal degeneration (CBD). Therefore, Myelin Basic Protein as the second most abundant myelin protein represents a promising target to explore.

Another significant feature of neurodegenerative diseases is neuroinflammation, which is also prevalent early in MSA and also in PD [5, 6]. This is evidenced by the presence of widespread microglial activation, which is a marker of neuroinflammatory changes [7]. However, different inflammatory markers in the CSF (e.g., YKL-40) have yielded inconclusive results in MSA [4]. Recent studies from the Alzheimer's field have highlighted the potential value of the soluble form of the triggering receptor expressed on myeloid cells 2 receptor (TREM2) as a promising biomarker candidate [8]. TREM2 is predominantly expressed by microglia and acts on the plasma membrane as a key member of the microglia sensome, mediating responses to several potential stimuli [9]. However, the value of soluble TREM2 as a marker of neuroinflammation in MSA and other atypical PD syndromes has not been evaluated before.

The current study therefore presents an analysis of CSF MBP and TREM2 in MSA as well as in atypical Parkinsonian conditions in two different cohorts. The findings contribute to the growing body of evidence indicating the importance of non-neuronal markers in neurodegenerative movement disorders.

## Methods

### Subjects

Biosamples were obtained from the movement disorder biobank of the Department of Neurology of the University Medical Center, Göttingen, Germany based on the availability of CSF samples independent of disease duration or disease severity. Patient samples were collected for the purpose of facilitating prospective research projects related to Parkinsonism. Control subjects (CTRL) were selected who did not display any clinical signs of neurodegeneration but who were comparable in age and gender characteristics. Patients with PD, Progressive Supranuclear Palsy (PSP), Corticobasal Syndrome (CBS) and Multiple System Atrophy (MSA-P, MSA-C) were diagnosed in accordance with acknowledged criteria [10–16]. Patients underwent neurological examination and history taking by movement disorder specialists. Assessment of motor function (UPDRS III) and cognitive function (MoCA or MMSE assessment) was available. If only MMSE was available, conversion into MoCA Score was applied [17]. All patients were included regardless of disease severity.

A validation cohort including PD patients, MSA patients and healthy controls were obtained from the Neuro-Biobank of the University of Tübingen, Germany. This biobank is supported by the local University, the Hertie Institute and the German Center for Neurodegenerative Diseases (DZNE). Age-matched controls were assessed to have no neurological diseases.

Permissions of the local ethics committees have been obtained prior to the initiation of the study (Ethics committee of the University Medical Center Göttingen, Nr. 13/11/12, 37/11/21; Ethics committee of the Faculty of Medicine at the University of Tübingen, Nr. 199/2011BO1). Written consent was provided by all patients or caregivers. The study conforms with the Code of Ethics of the World Medical Association (Declaration of Helsinki).

### CSF sampling

The collection of CSF samples was conducted in accordance with the established guidelines for the standardization of CSF biobanking [18]. As described before [19, 20], the initial 2–4 ml of CSF were withheld for routine analysis (WBC, RBC, total protein, total albumin), while the subsequent CSF were collected in polypropylene tubes. Samples were then centrifuged, aliquoted, and stored at − 80 °C within 1–2 h. Only samples with an RBC < 100/µl were used for analysis to exclude an influence due to blood contamination.

### Analyses of CSF proteins

The levels of total-tau and phospho-tau 181 were quantified using ELISA kits from Fujirebio (Fujirebio, Ghent, Belgium). These systems have been previously certified (CE-marked) for use in the clinical setting and are currently applied in our department. For the quantification of Myelin Basic Protein (MBP) and TREM2, commercially available ELISA kits were applied (MBP: AnshLabs, Texas, USA; TREM2: Abcam, Massachusetts, USA), assay metrics can be found in the supplement. Both kits have been validated by the manufacturer for the analysis of cerebrospinal fluid samples.

### Statistical analysis

The distribution of data was assessed visually (quantile–quantile plot) and by applying the Shapiro–Wilk normality test. Qualitative data were compared using the chi-squared test. For the comparison of group variables, either a one-way ANOVA or a non-parametric Kruskal–Wallis test was employed. Additionally, multiple regression was used for group comparison, adjusting for age, sex and disease duration. For the evaluation of significant regulations concerning CSF levels, MBP and TREM2 group comparison, Kruskal–Wallis with uncorrected Dunn’s test was used and PD as a control group was compared to all atypical Parkinsonian syndromes and controls, respectively. The Mann–Whitney test was applied for the evaluation of differences between the two groups. The correlation between the two variables was analyzed using Spearman’s rho and adjusted for age using multiple linear regression. The 95% confidence interval (CI) of the area under the ROC curve (AUC) was calculated according to the Wilson/Brown method. All analyses were performed using GraphPad Prism 9.4.1 or the R language 4.3.1.

## Results

### Subjects

MBP and TREM2 CSF levels were quantified in two independent cohorts (with a total of 171 samples). Cohort 1 consisted of predominantly early-stage patients (median disease duration of one year), while MSA in and PD patients in cohort 2 were more advanced (median disease duration of 4 years). Demographical characteristics can be found in Table 1. There were neither significant differences in age nor sex in cohort 1 and cohort 2, respectively.

**Table 1:** Demographical data of the study cohort

	Cohort 1 Göttingen					Cohort 2 Tübingen		
	CTRL *n* = 20	PD *n* = 20	MSA *n* = 20	DLB *n* = 13	PSP/CBD *n* = 23	CTRL *n* = 25	PD *n* = 25	MSA *n* = 25
Age, years	62 (43–83)	63 (42–77)	64 (43–83)	69 (42–77)	69 (53–85)	57 (40–81)	67 (43–76)	63 (47–76)
Male/female	10/10	10/10	11/9	9/4	14/9	14 / 11	14 / 11	14 / 11
Disease duration, years	NA	1 (1–12)	1 (1–6)	1 (1–7)	1 (1–4)	NA	4 (1–11)	4 (1–10)
UPDRS III	NA	21 (6–55)	31 (16–67)	20 (6–52)	26 (9–66)	NA	30 (10–58)	NA
MoCA	NA	27 (19–30)	25 (18–30)	17 (12–29)	21 (7–30)	NA	26 (12–30)	26 (15–30)

Values are presented as median (minimum to maximum)

CTRL, control; MOCA, Montreal Cognitive Assessment; UPDRS, Unified Parkinson Disease Rating Scale; PD, Parkinson’s disease; MSA, Multiple System Atrophy; DLB, Dementia with Lewy Bodies; PSP, Progressive Supranuclear Palsy; CBD, Corticobasal degeneration

### Quantification of CSF myelin basic protein

**Fig. 1:**
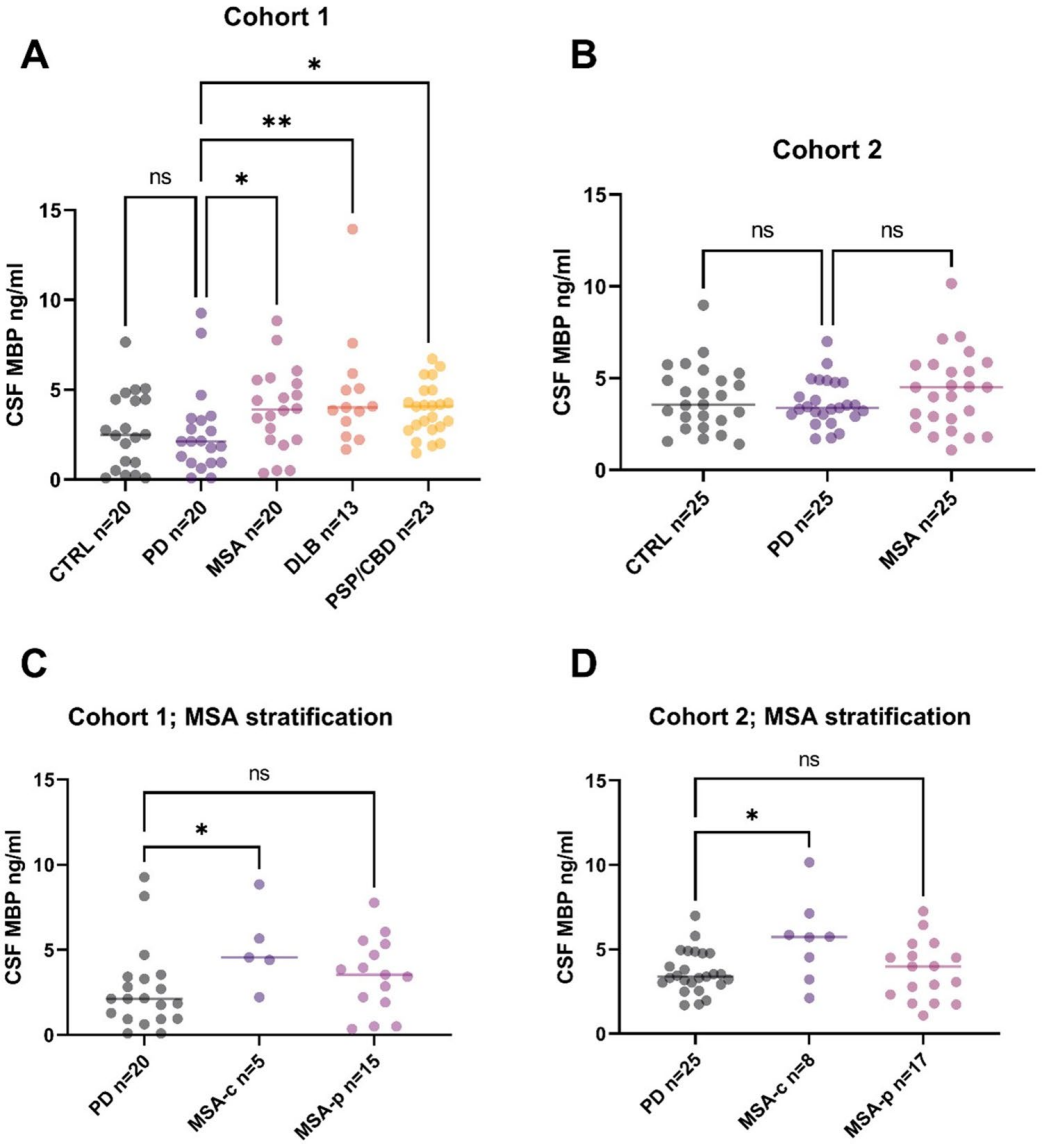
Quantification of CSF myelin basic protein (MBP) in two different cohorts. **a**, **b** Increased values can be demonstrated in atypical Parkinsonian syndromes compared to PD in cohort 1. The trend for increased values in MSA compared to PD in cohort 2 did not reach significance. **c, d** Upon stratification, MBP levels were found to be significantly higher in the MSA-c groups compared to PD in both cohorts

The results of the MBP analysis revealed a significant increase in CSF Myelin Basic Protein levels in all atypical Parkinsonian conditions compared to PD in cohort 1 (MSA vs. PD *p* = 0.019; DLB vs. PD *p* = 0.008; PSP/CBD vs. PD *p* = 0.012) (Fig. 1). There was no statistically significant difference between the PD and control subjects (*p* > 0.05). In cohort 2, no significant difference could be found between MSA and PD when the MSA subgroups were not stratified (*p* > 0.05). Upon stratification of MSA-p and MSA-c patients in both cohorts, it was observed that MBP levels were significantly higher in the MSA-c groups, respectively (cohort 1, *p* = 0.023; cohort 2, *p* = 0.033). When age, sex, and disease duration were taken into account through the application of multiple regression, significant intergroup differences between PD and the atypical conditions still remain evident (MSA vs. PD *p* = 0.046, DLB vs. PD *p* = 0.016, PSP/CBD vs. PD *p* = 0.029) in cohort 1.

### ROC-analysis applying myelin basic protein CSF levels

A ROC analysis was conducted to assess the discriminatory value of CSF MBP levels. The analysis revealed a significant discrimination between PD and MSA (*p* = 0.032, AUROC = 0.70), PD and DLB (*p* = 0.006, AUROC = 0.79) and PD and tauopathies (*p* = 0.006, AUROC = 0.74), respectively.

### Relationship between CSF myelin basic protein levels and clinical data

To assess the potential relationship between age and CSF MBP levels, Spearman correlation analysis was conducted using pooled disease groups and control subjects from cohort 1 and cohort 2. The results demonstrated that there was no significant correlation. The same analysis was performed on cohort 2, which also yielded no significant correlation (*p* > 0.05).

Upon analyzing disease subgroups, a significant inverse correlation was observed between MBP levels and MoCA cognitive scores (*p* = 0.035, *r* = − 0.44) in the PSP/CBD group in cohort 1, if not adjusted for multiple testing. Besides that, no correlation was found between clinical data (disease duration, UPDRS III, MoCA score) and MBP levels in relation to disease groups in cohort 1 and cohort 2, respectively (*p* > 0.05).

### Relationship between CSF myelin basic protein and CSF levels of neuronal degeneration markers

To assess the potential association between MBP and neuronal degeneration markers (Total tau protein levels and phospho-tau 181), Spearman correlation analysis was conducted. A highly significant correlation was demonstrated between total tau and MBP levels (*p* = 0.011, *r* = 0.29) in pooled disease groups, as well as between p-tau 181 and MBP levels (*p* = 0.002, *r* = 0.36) in cohort 1. This could not be reported for the pooled disease groups in cohort 2 (*p* > 0.05).

### Quantification of CSF TREM2

**Fig. 2:**
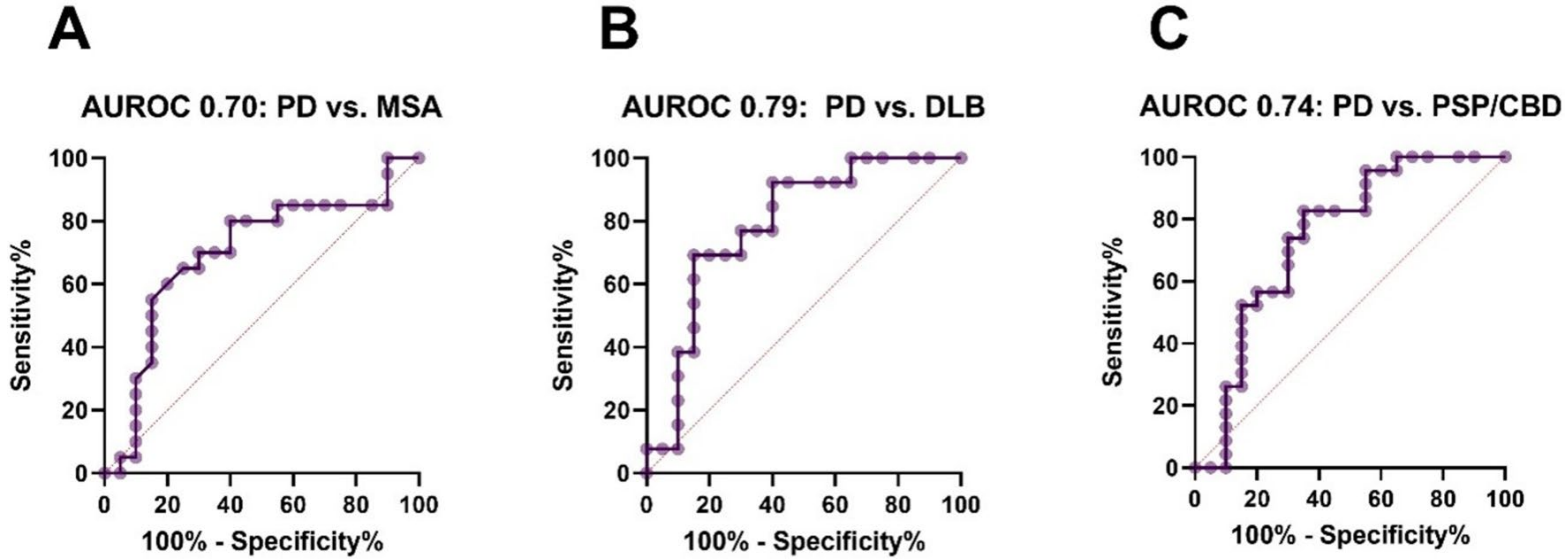
ROC curves for the discrimination of PD and MSA, DLB and PSP/CBD. Significant discrimination could be demonstrated for all comparisons

The results of the TREM2 analysis revealed no significant differences between the PD and the atypical Parkinsonian groups in both cohorts (*p* > 0.05) (Fig. 2). Given the established correlation between TREM2 CSF levels and age (as described below), an additional multiple regression model was constructed, adjusting for age and also for sex and disease duration as potential confounding variables. Here, significantly higher levels were demonstrated in the PD group compared to DLB in cohort 1 (*p* = 0.002). No significant differences were found in cohort 2 using this model.

### Correlation between CSF TREM2 levels and clinical data

To assess the potential relationship between age and CSF TREM2 levels, Spearman correlation analysis was performed using pooled disease groups and controls from cohort 1 and cohort 2. The results demonstrated a highly significant relationship between age and TREM2 in both cohorts (cohort 1: *p* = 0.0007, *r* = 0.34; cohort 2: *p* = 0.008, *r* = 0.38).

In cohort 1, there was also a significant correlation between TREM2 levels and the UPDRS III score in the PD group when not adjusted for multiple testing (*p* = 0.020, *r* = 0.53). Consequently, the correlation between TREM2 and UPDRS III could not be validated in cohort 2. There were no other significant correlations when considering other clinical data (disease duration, UPDRS III, MoCA score) along the disease subgroups. No significant correlations were found in cohort 2 (*p* > 0.05).

### Correlation between TREM2 and CSF levels of neuronal degeneration markers

A significant correlation was observed between total tau and TREM2 levels (*p* = 0.032, *r* = 0.25) in the pooled disease groups of cohort 1, as well as between phospho-tau and TREM2 levels (*p* = 0.0003, *r* = 0.42). As both variables correlate with age, multiple regression was employed to adjust for age as an important confounder. A significant relationship was still observed between p-tau181 and TREM2 levels (*p* = 0.027), whereas no significant correlation was found between tau and TREM2 levels (*p* = 0.74) in the subsequent analysis. In accordance, there was also a trend for a relationship between p-tau181 and TREM2 levels in the disease groups after adjusting for age (*p* = 0.066) in cohort 2.

## Discussion

The present study reports on the quantification of myelin basic protein (MBP) and soluble triggering receptor expressed on myeloid cells 2 (TREM2) in the CSF of a comprehensive movement disorder cohort. The primary hypothesis was that myelin damage and neuroinflammation could be more pronounced in MSA than in PD and in other Parkinsonian conditions. Accordingly, the present study concentrated on Multiple System Atrophy, while also encompassing samples from patients diagnosed with Parkinson's disease and additional atypical Parkinson syndromes, including Dementia with Lewy bodies (DLB) and Parkinson mimicking tauopathies (PSP and CBD). This approach allowed for a comprehensive comparison across different clinical entities.

In the early stages of MSA, pathological changes in myelin-associated proteins and morphological changes in oligodendrocytes (e.g., soma swelling) occur prior to pathological alpha-synuclein aggregation. To that end, Wenning and colleagues proposed a working model of MSA as a primary glial disorder (primary oligodendrogliopathy) with secondary neurodegeneration as a distinct disease entity [3]. Widespread degeneration of myelin can be found in MSA, while the total number of oligodendrocytes seems to be unaltered [21]. Together with proteolipid protein, myelin basic protein (MBP) constitutes the majority of the total myelin in the central nervous system. MBP is essential for the proper structural compaction of myelin layers where MBP stabilizes the major dense line to facilitate the adhesion of myelin layers [22].

The findings of elevated MBP levels in MSA compared to PD (Fig. 1) are consistent with the myelin-associated disease mechanisms as reported in MSA [3]. We hypothesize that this phenomenon reflects progressive myelin loss in MSA, which is attributed to the primary cause of the oligodendroglial pathology and to a lesser extent to the secondary consequence of the degeneration of myelinated axons. The higher MBP values observed in the cerebellar subtype of MSA compared to the Parkinsonian subtype in our study may indicate the presence of more distinct white matter pathology. This is potentially due to the olivopontocerebellar localization of the pathology in the MSA-c subtype. This is in line with recent studies employing multimodal imaging techniques have revealed more pronounced structural alterations in white matter tracts in MSA-c compared to MSA-p [23]. Given the limited sample size, the results of this sub-analysis should be interpreted with caution. To draw further conclusions, a multicenter approach with a larger number of patients is required.

To the best of our knowledge, MBP has not yet been evaluated in other common atypical Parkinsonian syndromes, namely DLB, PSP, or CBD. Interestingly, as with MSA, we also found higher MBP levels in all these atypical Parkinsonian conditions compared to PD (Fig. 1), which suggests the presence of a myelin and/or oligodendrocyte-associated pathology in these disorders. Compared to MSA, the evidence concerning white matter changes in these atypical Parkinsonian syndromes is relatively scarce. Nevertheless, there is some evidence in the literature to suggest that white matter damage is more pronounced in these diseases than in idiopathic Parkinson’s disease. Voxel-based analysis of fractional anisotropy using diffusion tensor imaging (DTI) revealed more severe white-matter abnormalities in patients with dementia with Lewy bodies (DLB) compared to controls and to patients with Parkinson’s disease dementia (PDD) with an involvement of the bilateral insular, bilateral posterior cingular, and bilateral visual association regions [24]. Proteomic studies have reported the dysregulation of multiple CSF proteins in DLB, with a significant enrichment in myelination processes [25]. Additionally, gene expression profiling of brain samples from patients with Lewy body dementia indicated a notable downregulation of genes associated with myelination [26]. Diffusion tensor imaging has also revealed widespread white matter abnormalities in cerebellar, brainstem, cerebral, and thalamic regions in PSP [27, 28]. A genome-wide association study has identified variants in the MOBP gene, which codes for a myelin-associated protein, also located in the major dense line of myelin. These variants have been shown to increase the risk of PSP. The authors concluded that myelin dysfunction therefore contributes to PSP pathogenesis [29]. In contrast to MBP, MOBP could not be detected over the limit of quantification in the current study applying a commercially available ELISA (data not shown) and therefore cannot be used as a biomarker applying this technique.

The evidence regarding the quantification of MBP CSF levels in MSA is limited. To the best of our knowledge, only one other group has assessed the CSF levels of Myelin Basic Protein, but this was not yet validated externally. In line with our results, they reported significantly higher CSF MBP levels in MSA compared to PD, but here without differences between MSA subtypes [30, 31]. The moderate discriminative value (AUROC 0.70–0.79, Fig. 2) of MBP indicates that it will not function as an individual biomarker for diagnosis. However, the longitudinal value is not yet known and there may be some value in monitoring disease progression, which needs to be investigated in further studies. Furthermore, this finding highlights the importance of the evaluation of non-neuronal disease mechanisms in MSA as potential diagnostic and therapeutic targets.

It is worth noting that cohort 2 did not include atypical Parkinsonian conditions besides MSA. This was based on our initial suspicion that we would only find MBP regulation in MSA, given the strong evidence in the literature indicating the involvement of myelin damage and oligodendrocyte dysfunction in the early disease stages [3]. We believe that further studies are needed to validate our finding of higher MBP levels in all atypical Parkinsonian conditions, as shown in our current trial.

**Fig. 3:**
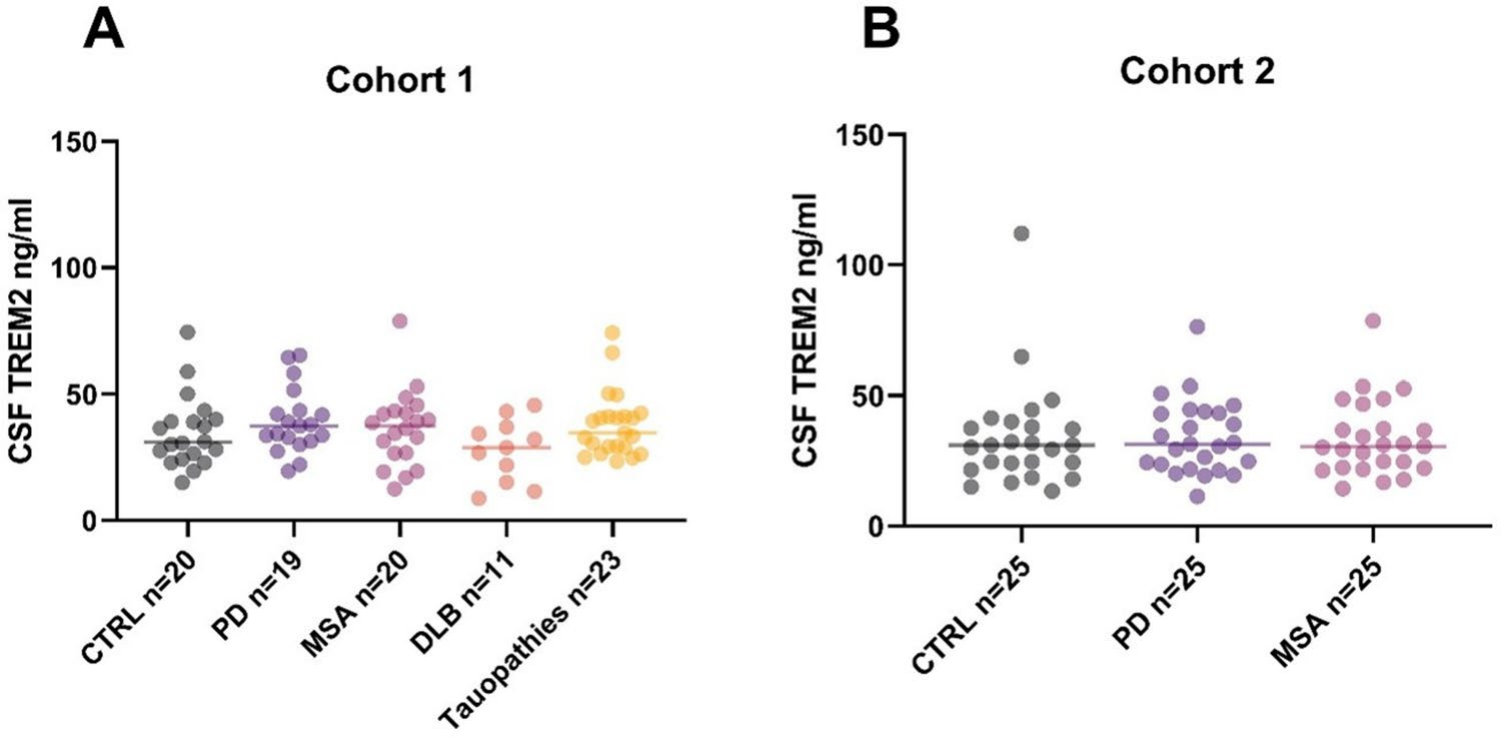
Quantification of TREM2 in two different cohorts. **a, b** There was no significant difference between PD and the atypical Parkinsonian in groups in both cohorts, respectively (Kruskal–Wallis test *p* > 0.05)

In addition to myelin dysfunction, neuroinflammation represents an important factor in the pathogenesis of MSA. An increased number of activated microglial cells has been observed in white matter regions [5]. Different biomarkers associated with neuroinflammation have been reported in the literature, including Flt3 ligand and YKL-40. However, these CSF biomarker candidates have shown no significant differences or even conflicting results in MSA [4]. In the field of Alzheimer’s disease, soluble TREM2 (a transmembrane protein abundantly expressed on microglia) presents a promising biomarker associated with neuroinflammation. Higher levels have been reported in the CSF of AD patients compared to controls, and TREM2 also correlates with tau and p-tau181 protein levels [32]. In Parkinson’s disease, TREM2 did not differ between healthy controls and patients with PD in the PPMI cohort [33]. Another group reported that CSF TREM2 concentrations were elevated in Parkinson's disease subgroups with a positive tau CSF biomarker signature, but not in Parkinson's disease subgroups with a positive CSF amyloid-β signature [34]. To the best of our knowledge, TREM2 has not yet been compared between different atypical Parkinsonian syndromes. In the present study, a significant difference was only identified between the PD and the DLB group if adjusted for age, sex, and disease duration (Fig. 3). We postulate that microglial activation is a more prominent feature in PD than in DLB. In line with this, it has been described that microglial activation is not a prominent feature of DLB [35]. Further validation in larger patient cohorts is necessary before any definitive conclusions can be drawn. The correlation between TREM2 and age has been described before and might reflect an enhanced expression of microglial TREM2, given that ageing is associated with increased microglial activity [36, 37]. With regard to the purported correlation between tau and TREM2 levels in PD, our findings also indicated a significant association between p-tau181 and TREM2 levels, even after adjusting for age using multiple regression. It is postulated that the absence of a correlation between disease duration and TREM2 levels may be attributable to the lack of precision in the data pertaining to the onset and duration of the disease, whereby first subtle symptoms may be overlooked and, therefore, not reported. However, it is also conceivable that a linear relationship may only be discernible at specific disease stages during the progression of the underlying neurodegeneration.

In conclusion, we report on elevated MBP but not TREM2 levels in the CSF of atypical Parkinsonian conditions compared to PD. A limitation of the current study is the lack of a reference standard for myelin damage, as no post-mortem tissue or diffusion tensor imaging (DTI) data (for microstructural myelin imaging) were available for the cohort. It is anticipated that additional longitudinal studies will be required to establish a proper correlation between the dynamics of myelin-associated biomarker targets and associated white matter changes.

## Conflicts of interest

The authors have no conflict of interest to report.

## Supplementary Information

Below is the link to the electronic supplementary material.

Supplementary file1 (DOCX 14 KB)

## Data Availability

The data supporting the findings of this study are available on request from the corresponding author.

## References

[1] Wenning GK, Fanciulli A (2014) Multiple system atrophy

[2] Kaji S, Maki T, Ishimoto T et al (2020) Insights into the pathogenesis of multiple system atrophy: focus on glial cytoplasmic inclusions. Transl Neurodegener 9:7 32095235 10.1186/s40035-020-0185-5 PMC7025408

[3] Wenning GK, Stefanova N, Jellinger KA et al (2008) Multiple system atrophy: a primary oligodendrogliopathy. Ann Neurol 64(3):239–246 18825660 10.1002/ana.21465

[4] Laurens B, Constantinescu R, Freeman R et al (2015) Fluid biomarkers in multiple system atrophy: a review of the MSA Biomarker Initiative. Neurobiol Dis 80:29–41 25982836 10.1016/j.nbd.2015.05.004

[5] Leńska-Mieciek M, Madetko-Alster N, Alster P et al (2023) Inflammation in multiple system atrophy. Front Immunol 14:1214677 37426656 10.3389/fimmu.2023.1214677 PMC10327640

[6] Tansey MG, Wallings RL, Houser MC et al (2022) Inflammation and immune dysfunction in Parkinson disease. Nat Rev Immunol 22(11):657–673 35246670 10.1038/s41577-022-00684-6 PMC8895080

[7] Kübler D, Wächter T, Cabanel N et al (2019) Widespread microglial activation in multiple system atrophy. Mov Disord 34(4):564–568 30726574 10.1002/mds.27620 PMC6659386

[8] Rauchmann B-S, Schneider-Axmann T, Alexopoulos P, Perneczky R (2019) CSF soluble TREM2 as a measure of immune response along the Alzheimer’s disease continuum. Neurobiol Aging 74:182–190 30458365 10.1016/j.neurobiolaging.2018.10.022 PMC6331262

[9] McQuade A, Kang YJ, Hasselmann J et al (2020) Gene expression and functional deficits underlie TREM2-knockout microglia responses in human models of Alzheimer’s disease. Nat Commun 11(1):5370 33097708 10.1038/s41467-020-19227-5 PMC7584603

[10] McKeith IG, Boeve BF, Dickson DW et al (2017) Diagnosis and management of dementia with Lewy bodies: fourth consensus report of the DLB Consortium. Neurology 89(1):88–100 28592453 10.1212/WNL.0000000000004058 PMC5496518

[11] Armstrong MJ, Litvan I, Lang AE et al (2013) Criteria for the diagnosis of corticobasal degeneration. Neurology 80(5):496–503 23359374 10.1212/WNL.0b013e31827f0fd1 PMC3590050

[12] Gilman S, Wenning GK, Low PA et al (2008) Second consensus statement on the diagnosis of multiple system atrophy. Neurology 71(9):670–676 18725592 10.1212/01.wnl.0000324625.00404.15 PMC2676993

[13] Höglinger GU, Respondek G, Stamelou M et al (2017) Clinical diagnosis of progressive supranuclear palsy: the movement disorder society criteria. Mov Disord 32(6):853–864 28467028 10.1002/mds.26987 PMC5516529

[14] Litvan I, Agid Y, Calne D et al (1996) Clinical research criteria for the diagnosis of progressive supranuclear palsy (Steele-Richardson-Olszewski syndrome): report of the NINDS-SPSP international workshop. Neurology 47(1):1–9 8710059 10.1212/wnl.47.1.1

[15] Hughes AJ, Daniel SE, Kilford L, Lees AJ (1992) Accuracy of clinical diagnosis of idiopathic Parkinson ’s disease: a clinico-pathological study of 100 cases. J Neurol Neurosurg Psychiatry 55:181–184 1564476 10.1136/jnnp.55.3.181 PMC1014720

[16] McKeith IG, Dickson DW, Lowe J et al (2005) Diagnosis and management of dementia with Lewy bodies: third report of the DLB Consortium. Neurology 65(12):1863–1872 16237129 10.1212/01.wnl.0000187889.17253.b1

[17] Lawton M, Kasten M, May MT et al (2016) Validation of conversion between mini-mental state examination and montreal cognitive assessment. Mov Disord 31(4):593–596 26861697 10.1002/mds.26498 PMC4864892

[18] Teunissen CE, Petzold A, Bennett JL et al (2009) A consensus protocol for the standardization of cerebrospinal fluid collection and biobanking. Neurology 73(22):1914–1922 19949037 10.1212/WNL.0b013e3181c47cc2 PMC2839806

[19] Maass F, Michalke B, Willkommen D et al (2021) Cerebrospinal fluid Iron-Ferritin ratio as a potential progression marker for Parkinson’s disease. Mov Disord 36(12):2967–2969 34553776 10.1002/mds.28790

[20] Maass F, Michalke B, Willkommen D et al (2020) Elemental fingerprint: Reassessment of a cerebrospinal fluid biomarker for Parkinson’s disease. Neurobiol Dis 134:104677 31733347 10.1016/j.nbd.2019.104677

[21] May VEL, Ettle B, Poehler A-M et al (2014) α-Synuclein impairs oligodendrocyte progenitor maturation in multiple system atrophy. Neurobiol Aging 35(10):2357–2368 24698767 10.1016/j.neurobiolaging.2014.02.028 PMC4087058

[22] Wong JH, Halliday GM, Kim WS (2014) Exploring myelin dysfunction in multiple system atrophy. Exp Neurobiol 23(4):337–344 25548533 10.5607/en.2014.23.4.337 PMC4276804

[23] Dash SK, Stezin A, Takalkar T et al (2019) Abnormalities of white and grey matter in early multiple system atrophy: comparison of parkinsonian and cerebellar variants. Eur Radiol 29(2):716–724 29974222 10.1007/s00330-018-5594-9

[24] Lee JE, Park H-J, Park B et al (2010) A comparative analysis of cognitive profiles and white-matter alterations using voxel-based diffusion tensor imaging between patients with Parkinson’s disease dementia and dementia with Lewy bodies. J Neurol Neurosurg Psychiatry 81(3):320–326 19828477 10.1136/jnnp.2009.184747

[25] Del Campo M, Vermunt L, Peeters CFW et al (2023) CSF proteome profiling reveals biomarkers to discriminate dementia with Lewy bodies from Alzheimer´s disease. Nat Commun 14(1):5635 37704597 10.1038/s41467-023-41122-y PMC10499811

[26] Pietrzak M, Papp A, Curtis A et al (2016) Gene expression profiling of brain samples from patients with Lewy body dementia. Biochem Biophys Res Commun 479(4):875–880 27666482 10.1016/j.bbrc.2016.09.114 PMC5079284

[27] Saini J, Bagepally BS, Sandhya M et al (2012) In vivo evaluation of white matter pathology in patients of progressive supranuclear palsy using TBSS. Neuroradiology 54(7):771–780 22160214 10.1007/s00234-011-0983-7

[28] Whitwell JL, Master AV, Avula R et al (2011) Clinical correlates of white matter tract degeneration in progressive supranuclear palsy. Arch Neurol 68(6):753–760 21670399 10.1001/archneurol.2011.107 PMC3401587

[29] Im SY, Kim YE, Kim YJ (2015) Genetics of progressive supranuclear palsy. J Mov Disord 8(3):122–129 26413239 10.14802/jmd.15033 PMC4572662

[30] Santaella A, Kuiperij HB, van Rumund A, Esselink RA, Bloem BR, Verbeek MM (2020) Cerebrospinal fluid myelin basic protein is elevated in multiple system atrophy. Parkinson Relat Disord 76:80–84 10.1016/j.parkreldis.2020.06.004 32576494

[31] Abdo WF, van de Warrenburg BPC, Kremer HPH et al (2007) CSF biomarker profiles do not differentiate between the cerebellar and parkinsonian phenotypes of multiple system atrophy. Parkinson Relat Disord 13(8):480–482 10.1016/j.parkreldis.2007.02.002 17448720

[32] Yang J, Fu Z, Zhang X et al (2020) TREM2 ectodomain and its soluble form in Alzheimer’s disease. J Neuroinflammation 17(1):204 32635934 10.1186/s12974-020-01878-2 PMC7341574

[33] Qin Q, Wan H, Wang D et al (2022) The association of CSF sTREM2 with cognitive decline and its dynamic change in Parkinson’s Disease: analysis of the PPMI Cohort. Front Aging Neurosci 14:892493 35783125 10.3389/fnagi.2022.892493 PMC9245456

[34] Wilson EN, Swarovski MS, Linortner P et al (2020) Soluble TREM2 is elevated in Parkinson’s disease subgroups with increased CSF tau. Brain 143(3):932–943 32065223 10.1093/brain/awaa021 PMC7089668

[35] Amin J, Holmes C, Dorey RB et al (2020) Neuroinflammation in dementia with Lewy bodies: a human post-mortem study. Transl Psychiatry 10(1):267 32747635 10.1038/s41398-020-00954-8 PMC7400566

[36] Henjum K, Almdahl IS, Årskog V et al (2016) Cerebrospinal fluid soluble TREM2 in aging and Alzheimer’s disease. Alzheimers Res Ther 8(1):17 27121148 10.1186/s13195-016-0182-1 PMC4848774

[37] Ana B (2024) Aged-related changes in microglia and neurodegenerative diseases: exploring the connection. Biomedicines 12(8):1737 39200202 10.3390/biomedicines12081737 PMC11351943

