# Supplementary material for: Myelin basic protein and TREM2 quantification in the CSF of patients with Multiple System Atrophy and other Parkinsonian conditions
Source: J Neurol. 2024 Dec 12;272(1):52. doi: 10.1007/s00415-024-12747-w (PMC11638341; doi:10.1007/s00415-024-12747-w)
Supplement: Supplementary file 1 — Supplementary file1 (DOCX 14 KB) [file 415_2024_12747_MOESM1_ESM.docx]

**Detailed MBP Elisa Kit characteristics according to the manufacturer can be found at:** (<https://www.anshlabs.com/wp-content/uploads/inserts/AL108.pdf>)

LOD = 0.093 ng/mL

**TREM2 Elisa Kit characteristics according to the manufacturer can be found at:** (<https://doc.abcam.com/datasheets/active/ab224881/en-us/human-trem2-elisa-kit-ab224881.pdf>)

LOD = 10.5 pg/mL
